# Effects of water and feed based RISCO-NUTRIFOUR probiotic supplementation on the technological and physicochemical quality of broiler breast meat

**DOI:** 10.3389/fvets.2025.1517078

**Published:** 2025-07-23

**Authors:** Abdulaziz A. Al-abdullatif, Maged A. Al-Garadi, Mohammed M. Qaid, Abdulkareem M. Matar, Mohsen M. Alobre, Mohammed A. Al-Badwi, Elsayed O. Hussein, Gamaleldin M. Suliman

**Affiliations:** Department of Animal Production, College of Food and Agriculture Sciences, King Saud University, Riyadh, Saudi Arabia

**Keywords:** broilers meat quality, feed conversion ratio, meat texture, RISCO-NUTRIFOUR probiotic, physicochemical properties, sensory attributes

## Abstract

**Introduction:**

Consumer interest in poultry and high-quality meat products has increased. Probiotics are used in the diet to improve the quality of broiler meat. The aim of this study was to investigate the effects of multi-strain probiotics (RISCO-NUTRIFOUR®, RNF) on the quality and physicochemical properties of broiler meat.

**Methods:**

A total of 288 broilers received six feed treatments for 1-14 days in water and 15-28 days in feed. T1-T3 received 0.4%, 0.2%, and 0.1% RNF, respectively; T4 received 0.1% *Bacillus subtilis* (BS; CLOSTAT®); T5 received 0.1% *Saccharomyces cerevisiae* (SC); and T6 received 0.0% probiotic (NC). The meat quality and physicochemical properties of the broiler breast were evaluated on day 28.

**Results and discussion:**

RNF, especially at 0.1% RNF, significantly reduced cooking losses was more tender (required the least SF), and improved average body weight at day 28 and total numerical feed conversion ratio compared to controls. The RNF probiotic had a positive effect on the texture profile (especially 0.4% RNF), sensory properties, and body weight (especially at 0.2% RNF). In conclusion, 0.4% RNF is recommended to achieve the best texture profile, 0.2% RNF to achieve the best juiciness and overall sensory acceptability as well as the best target weight of the broilers, and 0.1% RNF to achieve the most tender texture and the lowest cooking losses at day 28 compared to the controls.

## 1 Introduction

Chicken is the most popular poultry species and accounts for one third of the world’s meat production for human consumption, providing both meat and eggs (1). Chicken meat is an important source of high-quality protein and is in high demand worldwide due to its nutritional value and affordability (2, 3). As consumer preference shifts toward healthier diets, meat quality has become a critical factor in poultry production (4, 5).

The widespread use of antibiotics in poultry has raised concerns about antibiotic resistance, imbalance of the intestine microbiota, and the accumulation of residues in meat products. As a result, probiotics have emerged as viable, safe, and effective alternatives to antibiotics that promote gut health, immunity, and meat quality without undesirable side effects (1, 6). Among the various dietary supplements (7), probiotics such as *Bacillus subtilis* and *Saccharomyces cerevisiae* have shown potential to improve meat quality (5, 8). Probiotics have gained considerable attention in poultry nutrition as natural growth promoters and alternatives to antibiotics that improve gut health, feed efficiency, and overall meat quality.

Meat quality is significantly influenced by the microbial and physicochemical properties of the meat. Physicochemical properties, such as pH and color, and technological meat quality, such as water retention, drip loss, cooking loss, and shear force, are the most important and noticeable indices (1, 2, 9, 10). However, the effects of probiotics on these parameters are inconsistent across studies, and conflicting results are reported regarding their effects on broiler meat characteristics (4, 11–14).

This well-designed study addresses the demand for high-quality poultry meat by testing RISCO–NUTRIFOUR, a multi-strain probiotic (*Bacillus substiles*, *Lactobacillus Parabunchneri*, *Saccharomyces cerevisiae* yeast, *Lactobacillus harbinensis*, *Rhodopseudomonas palustris*, *Rhodopseudomonas shaeroides*, and *Candida ethanolic*) and its effects on meat quality parameters such as pH, color, water-holding capacity, cooking loss, texture, and sensory properties. The effects of multi-strain probiotics, such as RISCO-NUTRIFOUR® (RNF) on the physicochemical and technological quality of broiler meat, especially when administered by different routes, continue to be the subject of active research. The aim of this trial was to evaluate the effects of RNF probiotic supplementation-administered in water from day 1 to 14 and in feed from day 15 to 28-on the physicochemical properties, technological meat quality parameters, texture profile analysis (TPA), and sensory attributes of broiler breast meat, carcass characteristics, and feed conversion ratio of broiler chickens.

## 2 Materials and methods

### 2.1 Housing birds and experimental design

This study was conducted in June 2022 at King Saud University (KSU) Experimental Poultry Research Unit using 288 day-old Ross 308 chicks. The experiment complied with all applicable methods and procedures approved by the KSU Scientific Research Ethics Committee under the institutional approval code KSU-SE-21-47. The chicks were separated according to feather sex, weighed individually, and randomly divided equally into 6 experimental groups. Each treated group had 8 replicates with 6 birds each (3♂ and 3♀) (48 chicks per group). Experimental groups 1–3: groups treated with RNF at the three RNF doses 1, 2, and 3 (0.4, 0.2, and 0.1% RNF, respectively). Group 4: group treated with *Bacillus substiles* (BS, Clostat®). Group 5: group treated with *Saccharomyces cerevisiae* yeast (SC). Group 6: non-treated group (negative control).

The study investigated the effects of RISCO–NUTRIFOUR probiotic supplementation using two different administration methods: Water supplementation from 1 to 14 days and feed supplementation from 15 to 28 days. Supplementation in drinking water ensures a steady intake, especially in the first two weeks when feed intake is still in progress and the digestive system is not yet mature, from the 15th onwards, as the digestive system matures and feed intake increases, nutrient absorption becomes more efficient through feed. The broilers were treated with 1 of six water–based (from 1 to 14 days) and feed–based (from 15 to 28 days) treatments: 4 L/ton (0.4%), 2 L/ton (0.2%), 1 L/ton (0.1%), 0.1% Clostat “1:128,” 0.1% SC, and negative control (NC) 0%. Al Raya Specialties Industrial Factory in Riyadh, Saudi Arabia, manufactures the RISCO-NUTRIFOUR® solution, a probiotic mixture. RNF contains *Bacillus substiles* (1 × 10^^9^ colony-forming units (CFU)/ml), *Lactobacillus Parabunchneri* (1 × 10^^9^ CFU/mL), *Saccharomyces cerevisiae* yeast (1 × 10^^5^ CFU/mL), *Lactobacillus harbinensis* (1 × 10^^9^ CFU/mL), *Rhodopseudomonas palustris* (1 × 10^^7^ CFU/mL), *Rhodopseudomonas shaeroides* (1 × 10^^7^ CFU/mL), and Candida ethanolic (1 × 10^^5^ CFU/mL).

The experimental starter (0–14 days) and grower (15–28 days) diets were formulated as a mash according to the nutritional requirements of the Ross 308 Management Guide recommendations (Aviagen, 2019, New York, NY, USA), as shown in Supplementary Table S1. Feed and water were provided *ad libitum*. Chicks were housed in electrically controlled heated battery cages, with a room temperature of 35°C on arrival and a gradual decrease (2° C every 3 days) until day 24. The average outside temperature was approximately 26.4°C, and humidity ranged from light to moderate. The light bulb program was on continuously for 24 h during the first week of life and was on 23 h and off for 1 h during the rest of the experimental period. The broilers were housed in the same cage, which was 58 cm long, 50 cm wide, and 35 cm high. The stocking density was 6 birds per 0.30 m^2^. All chicks were immunized against Gumboro disease, Newcastle disease, and infectious bronchitis (Fort Dodge Animal Health-USA).

### 2.2 Bioactive chemicals analysis of RISCO–NUTRIFOUR

The procedures for the separation of chemical mixtures by gas chromatography–mass spectrometry (GC–MS; Agilent Technologies, Palo Alto, CA, USA) was described in detail by Azzam et al. (15). The bioactive chemicals were expressed as a proportion of the extracted samples.

### 2.3 Carcass traits, target weight, feed conversion ratio, and meat quality evaluation

The weights of the chicks at arrival and at the end of the trial were converted to the average target weight and used to calculate the daily weight gain. Then, the feed conversion ratio (1–28 d) was calculated by dividing the feed intake by the gain throughout these periods. On day 28, the broilers were slaughtered according to standard practice in Saudi Arabia, and eight male broilers per group (*n* = 8) were randomly selected to evaluate meat quality and carcass traits. The slaughter of broilers according to Islamic law complies with halal standards, with an emphasis on humane and respectful treatment during the slaughter process without anesthetic. The slaughtering was done with a very sharp knife and by a qualified person to allow for a faster process while minimizing the suffering of the birds, which is critical under halal standards, with an emphasis on maintaining the cleanliness and consistency of the meat. Slaughter weight and carcass weight (excluding head, neck, feathers, shanks, abdominal fat, and eviscerated organs) were measured. The carcass yield (%) was calculated as the ratio between carcass weight and slaughter weight. Breast, leg, wing, kidney, pancreas, lymphoid organs (liver, bursa, spleen, and thymus), and offal (gizzard, proventriculus, heart, and liver without gall bladder) were separated and weighed in the same manner. The percentage yield for each portion was estimated in relation to the live weight at slaughtering (16). After cutting, samples of the pectoral muscle were taken from each carcass to determine the physicochemical parameters (pH and color) and samples of the pectoralis major muscle to determine the textural characteristics and sensory properties. The samples were stored in the refrigerator at 4°C for 24 h after slaughter to measure the ultimate physicochemical parameters and then immediately stored at −20°C to determine the meat quality parameters. The frozen samples were thawed at 4°C before being tested for water-holding capacity (WHC), cooking loss (CL), myofibrillar fragmentation index (MFI), shear force (SF), texture profile analysis (TPA), and sensory evaluation.

### 2.4 Physico-chemical properties (pH value, core temperature, and color measurements)

The internal temperature and pH parameters of the pectoral muscles were determined 15 min and 24 h post-mortem using a thermocouple thermometer, taking the average of three pH measurements on the inner surface of the pectoral muscles at different locations for each sample. The pH was measured by inserting electrodes into the meat samples using a Hanna Instruments pH meter with microprocessor (model pH 211, Woonsocket, RI, USA).

The color parameters (L*, a*, and b* values and their derivatives) of the breast samples were measured 24 h post-mortem, whereby the average of two color measurements on the inside of the breasts was determined for each sample. Breast muscle color measurements were taken using a Minolta Chroma-Meter (Konica Minolta, Tokyo, Japan) with a CR400 head at an illumination setting compatible with D_65_ illumination (17). The coordinates L*, a*, and b* were evaluated according to the CIELAB system, where L* corresponds to lightness, a* to redness (between green and red), and b* to yellowness (between blue and yellow). The measurements were made after calibrating the device with a white reference tile at *Y* = 86.10, *x* = 0.3188, and *y* = 0.3362.

The center of the plane is neutral, and the distance from the center axis represents the color saturation “chroma,” while the angle on the chromaticity axes refers to the hue angle (18). In order to obtain a particularly realistic assessment of how consumers imagine the color of meat, chroma, delta color change (∆E), browning index (BI), whiteness index (WI), and hue angle (h*) were derived from the color coordinates and calibration values and formulated as described by Valizadeh et al. (19) and Cázares-Gallegos et al. (20).

### 2.5 Meat quality indicators

To measure water-holding capacity (WHC), the compression method described by Wilhelm et al. (21) was used. Thawed samples with a wet weight of about 2 to 3 g were carefully clamped between two sheets of filter paper and pressed for 5 min with a pressure device over two acrylic plates with a force of 10 kg; the samples were weighed again. The samples were analyzed in duplicate. Finally, the percentage of WHC was determined using the following equation: WHC (%) = 100 – [((Initial weight of sample – Final weight of sample/Initial weight of sample)) × 100]. Cooking loss (CL) is a common method for evaluating the water-holding capacity of meat during cooking. It is calculated as the percentage weight loss during the cooking process as described by Hussein et al. (22). In brief, the breast meat samples were weighed raw (initial weight, W₁). Samples are placed on a standard tabletop grill preheated to a specific temperature (e.g., 170–180°C). The samples are cooked until they reach an internal temperature of 75°C, which is recommended for poultry meat. The internal temperature is measured with a thermometer inserted into the thickest part of the breast. After cooking, the samples are cooled to room temperature (~20–25°C). The cooked samples are weighed again (final weight, W₂). To calculate the cooking loss (%):


$$\mathrm{CL}\;(\%)=\frac{\left(W1-W2\right)}{W1\;}*100$$

Where: W₁ = Initial raw weight (g), W₂ = Final cooked weight (g).

The MFI of muscle samples was measured as an indirect indicator of calpain activity using the method described by Suliman et al. (23). Thawed, scissor-cut samples (4 g) were homogenized in 40 mL MFI buffer (2°C) for 30 s using a blender. After washing several times with MFI buffer, the absorbance was measured at 540 nm using a spectrophotometer (HACH DR/3000, Loveland, CO, USA). The MFI was calculated by multiplying the absorbance of the resulting 0.5 mg/mL solution by 200. To calculate the shear force (SF) as an index of breast meat tenderness, five rectangular core samples of 2^*^1^*^1 cm^3^ in size from each chilled, cooked sample were cut longitudinally parallel to the muscle fibers using a manual corer. The greatest force (N/cm^2^) of the TA-HD Texture Analyzer (Stable Micro Systems Ltd., Godalming, UK) equipped with a Warner-Bratzler shear barb with a triangular opening blade, could be applied vertically to the fibers. The crosshead was configured to move at 200 mm/min. From a distance of 15 mm, the device was operated at speeds of 2, 2, and 10 mm/s during the pre-, during-, and post-tests. The SF values were calculated using the maximum point of the generated curve.

### 2.6 Texture profile analysis (TPA)

The TPA was performed with a TA-HD Texture Analyzer. To determine the TPA, the cooked breast muscle fibers were scored parallel to the longitudinal direction using a hand-held coring device. A cylindrical piston was used to compress the samples to 80% of their original height over two test cycles. The force-time curves of the deformation were determined using test-specific analyzes in the texturometer. The velocities used were 2, 5 and 5 mm/s in the pre-, intermediate and post-test. The hardness, springiness, cohesiveness, and chewiness of the samples were measured as described by Novaković and Tomašević (24).

### 2.7 Sensory evaluation

The frozen meat samples were thawed overnight at 4°C, then wrapped in aluminum foil and cooked in the oven at 200°C until a core temperature of 70°C was reached. After cooking, the samples were cut into small pieces of approximately 2 cm^3^ and given a random code number for identification. Twenty-four trained KSU taste panelists were asked for a sensory evaluation of the meat. The mean of all panel ratings was calculated to determine the characteristics of the sample. The evaluation was carried out according to the method described by Grunert et al. (25) using a 9-point hedonic scale, whereby the meat samples used for the sensory evaluation were divided into the following groups based on the category scaling: 9, 8, 7, 6, 5, 4, 3, 2, 1 = extremely like, very like, moderately like, somewhat like, neither like nor dislike, somewhat dislike, moderately dislike, very like dislike, extremely dislike, respectively. Water and crackers were served to remove any residual taste in the mouth from the previous samples.

### 2.8 Statistical analysis

The Ryan-Einot-Gabriel-Welsch and Quiot (REGWQ) test, also known as the “Ryan’s method,” is used to determine statistically significant differences (*p* < 0.05) between independent treatment groups in a balanced 1-way ANOVA reporting means ± standard error of the mean (SEM) based on a completely randomized design using the general linear model (GLM) of SAS (26) software (Cary, NC, USA).

The equation of the model was:


$$\gamma_{\mathrm{ij}}=\mu +T_{i}+e_{\mathrm{ij}}$$

Where *Y_ij_* is an individual observation, μ is the overall mean, *T_i_* is the effect of the *i*^th^ treatment, and *e*_ij_ is the random residual error. Before starting the statistical analysis, the Kolmogorov–Smirnov test was performed to ensure that the data were normal.

For the statistical analysis, a typical experimental design was established for this study, which included multiple treatment groups with standardized protocols for humane slaughter and random sampling, and data collection with a sufficient number of birds (8 per group) for the evaluation of carcass traits and meat quality. Six chicks per cage (8 cages per group) were used to evaluate growth performance.

## 3 Results

### 3.1 Physico-chemical traits, meat quality, texture profile analysis, and sensory evaluation of the breast

The data on the physicochemical parameters the breast meat of 28-day-old broilers treated with RNF in water from 1 to 14 days and with feed from 15 to 28 days are presented in Table 1. The treatments had no significant effect (*p* > 0.05) on pH 15 min, core temperature 24 h post-mortem, ultimate color components and color derivatives. However, 0.4% RNF had a significantly higher ultimate breast pH (pH_24_ h) compared to the other treatment groups.

**Table 1:** Initial and ultimate pH, core temperature, color components and color derivatives at day 28 in breast meat of broiler treated with RISCO–NUTRIFOUR probiotics in a water and feed bases.

Treatments^1^	RNF			0.1% *Bacillus subtilis*	0.1% *Saccharomyces cerevisiae*	Negative control	Standard error	*p* value
Item	0.4%	0.2%	0.1%					
Physicochemical properties								
Initial and ultimate pH								
Initial pH	6.19	6.11	6.20	5.97	6.08	6.07	0.062	0.109
Ultimate pH	6.08^a^	5.89^b^	5.92^b^	5.79^b^	5.86^b^	5.83^b^	0.039	0.0001
Core temperature	16.1	15.8	15.7	16.0	16.3	15.9	0.158	0.138
Ultimate color components and their derivatives								
Lightness (L*)	48.0	46.0	50.4	47.2	47.7	45.4	1.14	0.055
Redness (a*)	4.34	6.53	4.40	4.79	5.24	5.24	0.614	0.151
Yellowness (b*)	18.2	17.5	18.3	16.7	16.6	16.9	0.665	0.293
∆E	48.5	50.5	46.4	48.9	48.5	50.8	1.03	0.053
Chroma	18.8	18.9	18.9	17.4	17.5	17.7	0.634	0.261
Hue angle	76.3	69.6	76.0	74.1	72.5	72.7	2.00	0.195
Browning index	53.7	57.8	50.9	50.5	50.3	54.2	2.26	0.159
Whiteness index	44.7	42.8	46.8	44.4	44.8	42.5	1.01	0.053
a/b ratio	0.245	0.378	0.254	0.288	0.316	0.313	0.039	0.206

^1^Basal diet with 0.4, 0.2, and 0.1% RISCO–NUTRIFOUR (RNF), or with 0.1% *Bacillus subtilis*, or with 0.1% *Saccharomyces cerevisiae*, or without probiotics (Negative control). Each mean is based on measurements from 8 birds per treatment at 28 days of age. a-b Means in the same row with different superscripts differ significantly (*p* < 0.05). ∆E: total ultimate color change; Chroma: Saturation index; a*/b* ratio: redness to yellowness ratio.

The meat quality characteristics for the breast samples at 28 days of age are shown in Table 2. CL% and SF of the breast samples differed (*p* < 0.05) between treatments. The 0.1% RNF treated group had the lowest CL% and SF values, indicating that the 0.1% RNF treated group had the most favorable CL% (8.42%) and tenderness (the lowest tenderness; 3.42). Although WHC% and MFI were similar between groups (*p* > 0.05), the 0.1% RNF treated group had the highest numerical (*p* > 0.05) from WHC and MFI, which decreased with increasing RNF dosage.

**Table 2:** Meat quality characteristics on day 28 of age in breast meat of broilers treated with RISCO–NUTRIFOUR probiotics in a water and feed base.

Treatments^1^	RNF			0.1% *Bacillus subtilis*	0.1% *Saccharomyces cerevisiae*	Negative control	Standard error	*p* value
	0.4%	0.2%	0.1%					
WHC (%)	66.7	67.7	68.5	66.6	65.9	66.7	1.17	0.636
CL (%)	26.8^a^	20.5^ab^	8.42^c^	12.3^bc^	29.3^a^	12.1^bc^	2.51	<0.0001
MFI	103.4	105.4	112.7	102.2	105	108.5	5.47	0.779
SF (N)	4.21^b^	4.25^b^	3.42^c^	4.40^ab^	4.92^a^	4.35^ab^	0.193	0.0002

^1^Basal diet with 0.4, 0.2, and 0.1% RISCO–NUTRIFOUR (RNF), or with 0.1% *Bacillus subtilis*, or with 0.1% *Saccharomyces cerevisiae*, or without probiotics (Negative control). Each mean is based on measurements from 8 birds per treatment at 28 days of age. ^a–c^Means in the same column with different superscripts differ significantly (*p* < 0.05). WHC, Water holding capacity; CL, Cooking loss; MFI, myofibril fragmentation index; SF, shear force.

The TPA for the broiler samples on day 28 are shown in Table 3. Hardness (N), springiness, and cohesiveness of the breast meat samples differed (*p* < 0.05) between the treatment groups. Hardness was higher in groups treated with 0.1% BS and 0.1% SC probiotics, lower in groups treated with the RNF-probiotic mixture, and intermediate in the negative control group.

**Table 3:** Texture profile analysis (TPA) on day 28 of age in breast meat of broilers treated with RISCO–NUTRIFOUR probiotics in a water and feed base.

Treatments^1^	RNF			0.1% *Bacillus subtilis*	0.1% *Saccharomyces cerevisiae*	Negative control	Standard error	P value
	0.4%	0.2%	0.1%					
Hardness (N)	5.09^b^	5.36^b^	5.01^b^	6.83^a^	7.26^a^	5.79^ab^	0.309	<0.0001
Springiness	0.91^a^	0.86^ab^	0.90^a^	0.78^c^	0.79^bc^	0.78^c^	0.019	<0.0001
Cohesiveness	0.66^a^	0.60^ab^	0.64^ab^	0.58^b^	0.60^ab^	0.58^b^	0.018	0.014
Chewiness	3.07	2.94	2.91	3.15	3.46	2.66	0.221	0.218

^1^Basal diet with 0.4, 0.2, and 0.1% RISCO–NUTRIFOUR (RNF), or with 0.1% *Bacillus subtilis*, or with 0.1% *Saccharomyces cerevisiae*, or without probiotics (Negative control). *n* = 8 samples per treatment. ^a–c^Means in the same column with different superscripts differ considerably (*p* < 0.05).

The RNF treatments, particularly at 0.4%, gave the most significant values for springiness and cohesiveness in the meat samples when compared to the negative control, with the 0.4% RNF treated group having the best values for springiness and cohesiveness. The chewiness values were similar between treatments (*p* > 0.05).

The sensory evaluation of the broiler samples at 28 days of age is shown in Table 4. Consumer ratings of tenderness and flavor were similar between treatments (*p* > 0.05), while juiciness and overall acceptability were highest in broilers treated with 0.2% RNF in water from 1 to 14 days of age and in feed from 15 to 28 days of age compared to the other groups.

**Table 4:** Sensory attributes on day 28 of age in breast meat of broilers treated with RISCO–NUTRIFOUR probiotics in a water and feed base.

Treatments^1^	RNF			0.1% *Bacillus subtilis*	0.1% *Saccharomyces cerevisiae*	Negative control	Standard error	P value
	0.4%	0.2%	0.1%					
Juiciness	6.08^ab^	7.00^a^	6.42 ^ab^	6.33 ^ab^	5.75^b^	6.00 ^ab^	0.26	0.03
Tenderness	6.42	6.75	6.83	6.25	5.75	6.33	0.27	0.086
Flavor	6.00	6.33	6.00	6.25	6.25	6.50	0.21	0.513
Acceptability	6.67^ab^	7.25^a^	6.84^ab^	6.76^ab^	6.17^b^	7.00^a^	0.195	0.01

^1^Basal diet with 0.4, 0.2, and 0.1% RISCO–NUTRIFOUR (RNF), or with 0.1% *Bacillus subtilis*, or with 0.1% *Saccharomyces cerevisiae*, or without probiotics (Negative control). *n* = 8 samples per treatment. ^a–c^Means in the same column with different superscripts differ considerably (p < 0.05).

### 3.2 Carcass traits, target weight, and feed conversion ratio

The carcass characteristics (live slaughter weight (g), hot carcass weight (g), carcass yield (%), and relative carcass composition weights (% to live weight)) of 28-day-old broilers treated with RNF in water from 1 to 14 days and with feed for 15 to 28 days are shown in Table 5. The live weight, carcass weight, carcass yield, and carcass composition of the broilers did not differ (*p* > 0.05) between the experimental groups.

**Table 5:** Carcass traits measured at day 28 of broilers supplemented with RISCO–NUTRIFOUR probiotics in a water and feed bases.

Treatments^1^	RNF			0.1% *Bacillus subtilis*	0.1% *Saccharomyces cerevisiae*	Negative control	Standard error	P value
Item	0.4%	0.2%	0.1%					
Live weight (Kg)	1.55	1.62	1.64	1.54	1.57	1.49	0.046	0.334
Carcass weight (g)	0.942	0.996	1.065	0.944	0.967	0.908	0.039	0.168
Carcass yield (%)	60.9	61.5	65.2	61.4	61.6	60.9	1.78	0.577
Percentage weights (g/100 g BW of the broilers)								
Breast	28.2	28.3	28.7	28.2	27.1	29.9	0.802	0.372
Leg	24.6	24.4	24.5	25.9	25.5	25.8	1.50	0.350
Wing	5.25	4.49	4.92	5.27	5.12	4.98	0.857	0.494
Thymus	0.367	0.404	0.333	0.314	0.371	0.476	0.046	0.088
Bursa	0.216	0.204	0.252	0.217	0.161	0.224	0.021	0.286
Spleen	0.081	0.068	0.084	0.081	0.074	0.088	0.137	0.558
Kidney	0.502	0.531	0.547	0.457	0.578	0.509	0.031	0.372
Pancreas	0.268	0.290	0.276	0.273	0.293	0.300	0.401	0.559
Giblets	5.75	5.86	5.72	5.57	6.01	5.49	0.094	0.741

^1^Basal diet with 0.4, 0.2, and 0.1% RISCO–NUTRIFOUR (RNF), or with 0.1% *Bacillus subtilis*, or with 0.1% *Saccharomyces cerevisiae*, or without probiotics (Negative control). *n* = 8 samples per treatment. ^a–c^Means in the same row with different superscripts differ significantly (p < 0.05). ^2^Giblets (Gizzard, Proventriculus, heart, and liver without gallbladder).

**Figure 1:**
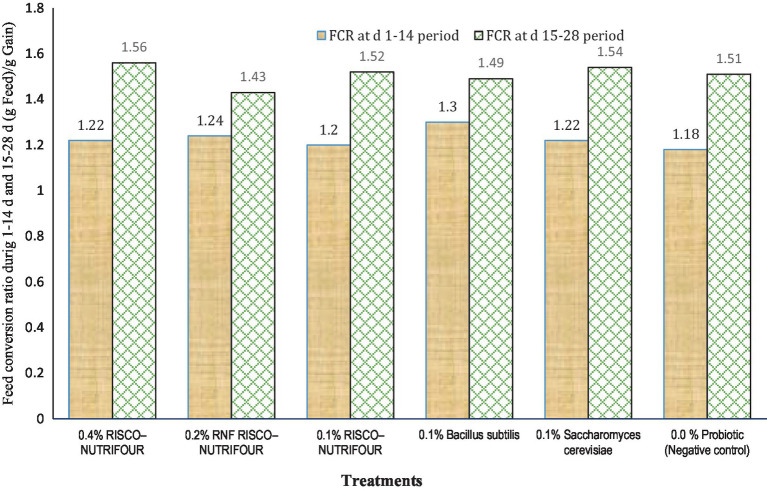
Feed conversion ratio (FCR) during 1–14 days’ period (*p* = 0.225; SEM ± 0.394); FCR during 15–28 days’ period (*p* = 0.394; SEM ± 0.042); *n* = 8 replicated cages.

**Figure 2:**
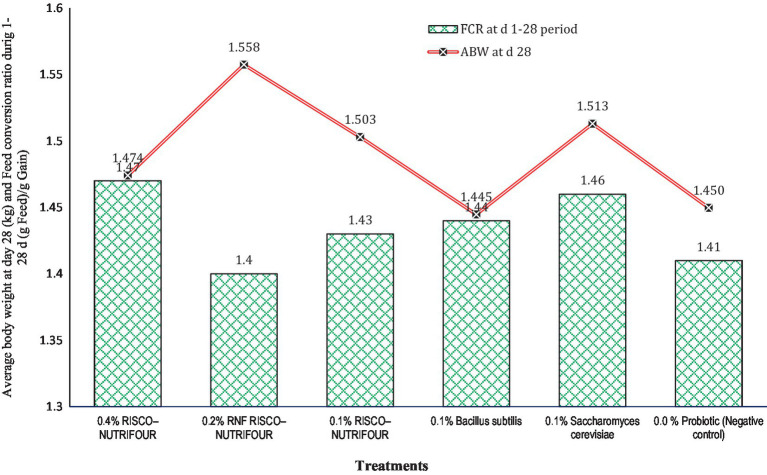
Average body weight at day 28 (kg; *p* = 0.041; SEM ± 0.027) and feed conversion ratio during 1–28 days’ periods (*p* = 0.041; SEM ± 0.027; FCR = feed intake (g)/gain (g)); *n* = 8 replicated cages.

Figure 1 shows the FCR of broilers treated with RNF in water for 1–14 days and with feed for 15–28 days. Water supplementation with RNF resulted in a lower (*p* > 0.05) FCR in broiler chicks compared to the negative control. However, FCR improved quantitatively between days 15 and 28, indicating that treatments with water supplementation may not be as effective in achieving optimal results. During the 1–14 days, 0.2% RNF treatment reduced FCR by 5.08% compared to the negative control (1.24 vs. 1.18). During 15–28 days, 0.2% RNF treatment improved (*p* > 0.05) FCR by 5.30% compared to negative control (1.51 *vs* 1.43). Thus, RNF was more effective when administered via feed rather than water compared to the control. The average body weight at day 28 and the overall FCR of broilers receiving RNF in water from 1 to 14 days and in feed from 15 to 28 days are shown in Figure 2. The 0.2% RNF treatment resulted in a significantly (*p* < 0.05) higher body weight (1.558 kg) on day 28 and a trend toward better feed conversion (1.40) throughout the period compared to the other treatments.

## 4 Discussion

Probiotics, live non-pathogenic microorganisms added to human and animal diets, colonize the intestinal environment to promote a balanced flora consisting of species commonly found in the poultry gut (27–30). Probiotics have the ability to reduce pathogens, and improve the quality of broiler meat (30–32). The effectiveness of probiotics in exerting beneficial effects depends on their ability to colonize the intestine, which is influenced by various factors. These include the feeding program, the type, dose, and frequency of probiotic administration, the presence of prebiotics, and host-related factors such as age, health status, genetic characteristics, the pH of the intestine. In addition, external environmental conditions play a crucial role in determining probiotic colonization and functionality (30, 33–35).

In this study, the effects of probiotic supplementation on the meat quality of broilers were investigated. Meat quality parameters such as pH, lightness, redness, yellowness, cooking loss, water holding capacity, shear force, texture profile, and sensory evaluation were assessed. Analysis of REGWQ showed that the physicochemical data of the breast meat at day 28 were similar in the RNF groups, except for the ultimate pH, which was higher in the highest RNF group compared to the other groups. The pH was considered a general signal for meat quality testing, reflecting the conversion of glycogen to lactic acid in the muscle pre and post mortem (9). At that time, there was a direct relationship between pH and meat quality, including tenderness, water-holding capacity, color, juiciness, and shelf-life. Meat generally had a pH between 5.0 and 7.0 (36). Some of the studies that examined meat quality found that the use of probiotics increased redness and yellowness of breast meat and decreased lightness (5, 37), while probiotics increased all parameters of meat color in thigh meat (13, 38). In contrast, some studies found that probiotic supplementation had no effect on yellowness, redness, or lightness (5, 13, 37, 39). The addition of probiotics has been shown to consistently improve the redness and yellowness of broiler meat, which could be an indication of improved meat quality as perceived by the consumer. These differences between trials were more significant for thigh meat than breast meat, indicating that probiotics had a different impact depending on the anatomical location of the muscle.

This study showed that the use of RNF probiotics in broilers significantly improves meat quality. The 0.4% RNF improved the breast texture profile of the breast through improved springiness by 16.67% indicating better elasticity and resilience of meat after compression, and improved cohesiveness by 13.79% compared to the negative control, indicating improved structural integrity and firmness of the meat, resulting in better meat texture.

The 0.2% RNF showed a 21.7% increase in juiciness, increasing meat palatability and consumer satisfaction, and a 17.5% increase in overall acceptability, indicating a higher consumer preference for RNF-treated meat, compared to the SC group, and a 7.45% increase in average body weight at day 28 compared to the negative control, indicating improved growth performance with the 0.2% RNF supplementation. Also, the lowest (best) FCR was observed at RNF 0.2%, indicating better feed efficiency compared to the other groups. The lower FCR value indicates that the broilers fed 0.2% RNF utilized the feed more efficiently, resulting in higher weight gain per unit of feed consumed.

The 0.1% RNF showed a 71.3% reduction in cooking loss, which improved water-holding capacity, as well as a 30.5% reduction in shear force and 30.99% reduction in hardness, which improved meat tenderness compared to the SC group at day 28. Thus, the study shows that RNF supplementation significantly improves meat quality, especially in terms of water retention, tenderness, and sensory properties. Tang et al. (5) found that dietary supplementation with BS can improve meat quality and carcass characteristics of broilers, which is beneficial to consumers due to the improved fatty acid profile and amino acid composition. Other parameters studied were not significantly altered by the treatments. Previous research on probiotics in broilers has primarily focused on growth rather than meat quality, leaving a gap in the literature (40, 41). As a result, this study presents information about meat quality.

Similarly, studies on other meat quality metrics show that probiotic supplementation has no effect on pH, cooking loss, shear force, or drip loss of broiler meat (5, 13, 39). In addition, some studies found that probiotic supplementation increased pH and WHC in breast meat (42, 43) while reducing cooking loss, shear force, and drip loss in breast and thigh meat (13, 37, 38). The increase in WHC% and decrease in CL% in breast meat show that RNF probiotics, especially at the 0.1% level, can alter protein structures in muscle, improving their ability to bind moisture during cooking. This improves both the sensory properties of the meat and its nutritional value. The various research studies on the effect of adding probiotics to broilers vary widely, including the breed of chicken, the type of probiotic, the level and dosage, and the location of measurement. Therefore, a study was needed to further investigate these effects on meat quality indicators.

Several factors contribute to the heterogeneity of the study results. In some studies, measurements were taken on both the leg and the breast, while in others only the breast or the leg was examined. These differences highlight the complex relationships between probiotic supplementation and meat quality in broilers. This study provides a detailed assessment of the effects of probiotics on numerous meat quality traits in broilers. The study found that the addition of RNF probiotics, particularly at 0.1%, had a significant effect on meat texture profile, sensory characteristics, cooking loss, and shear force, all of which were significantly improved in the broiler breast meat. In addition, numerical improvements in WHC and MFI were observed in the breast meat portions of broilers receiving RNF at a low concentration (0.1%). The results have important implications for the chicken industry, particularly with regard to improving meat quality by optimizing feed formulation.

## 5 Conclusion

Based on the finding obtained in this study, the 0.4% RNF is recommended to achieve a 16.67% improvement in springiness and a 13.79% improvement in cohesiveness meat texture compared to the negative control. With 0.2% RNF, a 21.7% increase in juiciness and a 17.5% increase in overall acceptability compared to the SC group, and a 7.45% increase in average body weight at day 28 compared to the negative control and overall feed conversion compared to other groups is recommended. It is recommended that 0.1% RNF achieves a 71.3% reduction in cooking loss, improving water-holding capacity, a 30.5% reduction in shear force and a 30.99% reduction in hardness, improving meat tenderness compared to the SC group on day 28. The study thus shows that supplementation with RNF significantly improves meat quality, particularly in term of water retention, tenderness, and sensory properties, and points to avenues for further research and standardization in poultry production. These results also contribute to a better understanding of the role of RISCONUTRIFOUR probiotics in improving meat quality and meeting consumer demands for nutritious and high-quality poultry products.

## Data Availability

The original contributions presented in the study are included in the article/Supplementary material, further inquiries can be directed to the corresponding authors.
