# Supplementary material for: Effects of water and feed based RISCO-NUTRIFOUR probiotic supplementation on the technological and physicochemical quality of broiler breast meat
Source: Front Vet Sci. 2025 Jul 23;12:1517078. doi: 10.3389/fvets.2025.1517078 (PMC12327400; doi:10.3389/fvets.2025.1517078)
Supplement: Supplementary file 1 [file Table_1.docx]

# **Supplementary Tables**

**Supplementary Table S1.** Nutrient content and ingredients of the basal diets (%).

| **INGREDIENTS** | **Starter** | **Grower** |
| --- | --- | --- |
| Corn Grain | 53.92 | 55.51 |
| Soybean Meal 48 | 38.23 | 35.90 |
| Soybean Oil | 3.44 | 4.65 |
| Limestone | 1.54 | 1.39 |
| Common Salt | 0.38 | 0.38 |
| Vitamin Premix^1^ | 0.10 | 0.10 |
| Mineral Premix^2^ | 0.10 | 0.10 |
| DL-Methionine | 0.35 | 0.31 |
| L-Lysine HCL | 0.21 | 0.15 |
| L-Threonine | 0.14 | 0.10 |
| Mono Calcium Phosphate | 1.59 | 1.41 |
| Choline CL-60% | 0.003 | **-** |
| Total | 100.00 | 100.00 |
| Calculated nutrient, % |  |  |
| ME, kcal/kg | 3000 | 3100 |
| Crude protein, % | 23.0 | 21.15 |
| Non phytate P, % | 0.48 | 0.44 |
| Calcium, % | 0.96 | 0.87 |
| D. Lysine, % | 1.28 | 1.15 |
| Sulfur amino acids, % | 0.95 | 0.87 |
| Threonine, % | 0.86 | 0.77 |
